# Resistance of Polymeric Laminates Reinforced with Fabrics against the Growth of Delaminations

**DOI:** 10.3390/ma14237367

**Published:** 2021-11-30

**Authors:** Piotr Czarnocki

**Affiliations:** Institute of Aeronautics and Applied Mechanics, Warsaw University of Technology, 00-665 Warsaw, Poland; pecz@meil.pw.edu.pl

**Keywords:** reinforcement orientation, delamination resistance, fabric reinforcement

## Abstract

Dependence of the initiation values of the Strain Energy Release Rate, G_Ci_, on the orientation of the reinforcement direction α relative to the delamination front was investigated for two laminates of different interfacial ply arrangements. In the case of the first laminate, the delamination was located at the interface of the layers reinforced with symmetric fabric and unidirectional fabric. In the case of the second laminate, the delamination was located at the interface of layers reinforced with symmetric fabric. In both laminates, the orientation of fibers in the layers separated by the delamination differed by 45° regarding the warp directions. The investigations were carried out for Mode I, Mode II, and Mixed-Mode I/II (G_II_/G_I_ = 1 and G_II_/G_I_ = 1.7) loadings using hybrid beam specimens. The major problem appearing in the intended tests was the inevitable lack of symmetry in the xz and xy planes of the specimens and the resulting deformation and stress–strain couplings, causing undesired loading modes. To decrease these couplings, especially designed hybrid beam specimens were used. An auxiliary finite element analysis was performed to assess the remaining effects of the reduced couplings. To ascertain whether statistically significant differences between Gci values for different α occurred, the one-way analysis of variance supplemented by Levene’s test was carried out. The dependence of Gci on α was found out for both laminates. However, it was not equally strong, and it turned out that the loading mode and the interfacial ply were arrangement sensitive.

## 1. Introduction

**Figure 1:**
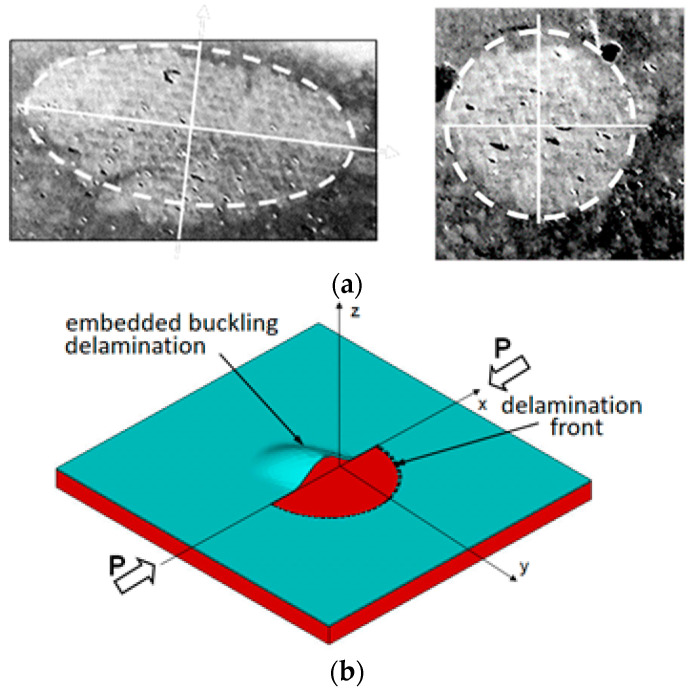
Delaminations: (**a**) white spots indicate the disbonds of the adjacent reinforcement layers which can buckle under compressive loading and propagate; (**b**) cross-section in the xz plane of an embedded buckling circular delamination under compressive loading.

Damage tolerance design accounts for already existing structural defects, e.g., delaminations, which are widespread laminate defects, as shown in Figure 1. Fracture mechanic tools can be applied to characterize the growth initiation and growth of such defects. Researchers can use (a) the critical value of the Strain Energy Release Rate (SERR), Gc, to determine the initiation of delaminations growth, and (b) the Paris law to assess the delamination growth rate. A typical laminate comprises a certain number of unidirectionally reinforced layers rotated relative to each other by θ to meet strength and stiffness requirements. The interfaces between the layers that differ by reinforcement orientation are particularly prone to delamination propagation. Therefore, the dependence of G_C_ on θ was broadly investigated, but mainly for laminates reinforced with unidirectional (UD) tapes. In [1], delamination resistance was investigated under Mode I, Mode II, and Mixed-Mode I/II (20% and 57% of Mode I) loadings for delaminations for 0°//0°, 22.5°//−22.5°, and 45°//−45° interfaces, and in [2] under Mode I and Mode II for 0°//0°, 30°//−30°, and 60°//−60° interfaces, and in [3,4] under Mode I and Mode II for 0°//0°, 30°//−30°, and 45°/−45° interfaces. Mode I delamination resistance was also investigated at 0°//0°, 15°//15°, and 30°//30° interfaces [5]. In [6], resistance against delamination was investigated for 0°//0°, 0°//45°, and 0°//90° interfaces under Mode I, and in [7] for 0°//0°, 0°//22.5°, 0°//45°, 0°//67.5°, and for 0°//90° under Mode II. In [8], resistance against delamination under Mixed-Mode loading (G_II_ = 34%, 85%) was investigated for 0°//0°, 0°//22.5°, 0°//45°, and 0°//90° interfaces. More recent results of similar investigations can be found, e.g., in [9,10,11,12,13,14,15,16,17,18], and in [19,20,21,22] where also R-curves were of interest.

**Figure 2:**
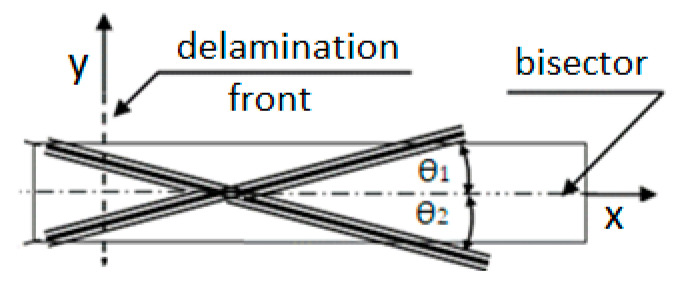
Arrangement of the reinforcement in the adjacent layers undergoing separation due to the delamination propagation. Relative orientation θ = θ_1_ + θ_2_ (θ_1_ = θ_2_) is referred to as interfacial ply orientation. Angles θ_1_ and θ_2_ can be measured relative to the specimen *x*-axis. The θ_1_ can be considered as an angle of reinforcement below the delamination plane, and θ_2_ can be considered as an angle of reinforcement above the delamination plane.

**Figure 3:**
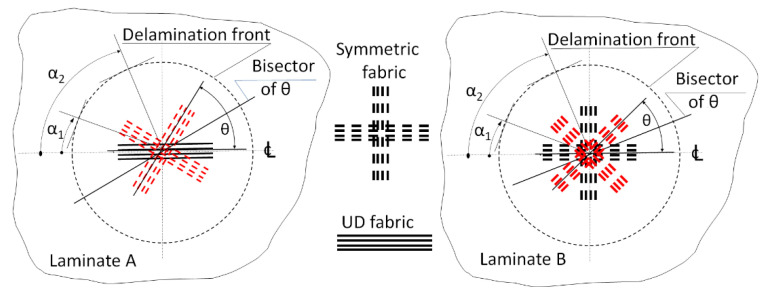
Variation of the reinforcement orientation relative to the front of buckling circular delamination.

The vast number of published papers focused on a particular delamination process in which a bisector of θ, (θ = θ_1_+ θ_2_), as shown in Figure 2, was perpendicular to a straight delamination front, e.g., [1,2,3,4]. In such a case, θ_1_ could be considered as an angle of reinforcement below and θ_2_ above the delamination plane, as shown in Figure 2. These angles define the interfacial ply orientations (IPO) θ. Delaminations of curvilinear fronts, e.g., embedded buckling ones, as shown in Figure 1, are of more practical significance than straight front delaminations because the former occurs in composite structures of practical use. In the case of such delaminations, the reinforcement direction relative to the direction of the delamination front, α, varies along the front, as shown in Figure 3, while the IPO remains unchanged. To the best of the author’s knowledge, papers dealing with such delamination processes are seldom, despite their practical importance. The only ones known to the author are [23,24]. Unfortunately, the mentioned papers consider UD tape reinforcement only.

Due to economic reasons, an application of fabric reinforcement is equally common as UD tapes because the former allows for a less laborious layup. A typical fabric reinforced laminate comprises a certain number of UD and symmetric fabrics or symmetric fabrics only, which are rotated relative to each other by θ = 45°. (In the case of fabric reinforcement, θ is the angle between the warps of the adjacent fabrics.) This research concerns resistance against delamination growth of such laminates and focuses on the G_ci_(α) relationship.

The reason for undertaking this research was the following. Very often, damage tolerance design must be supported by various numerical analyses. If a delamination growth is of interest, two pieces of information are needed. These are (a) initiation value of the Strain Energy Release Rate G_ci_(α) relationship and (b) propagation rate da/dn (n, α), which can be described by the Paris law. The presented research concerns the former because it is crucial and must be obtained in the first place. Furthermore, despite this need, there are few papers with public access concerning the G_ci_(α) relationship.

Numerical analysis concerning the distribution of the SERR components along a front of embedded buckling delamination [25] shows that Mode I and Mode II loadings prevail, while Mode III loading can be neglected. Therefore, the presented work was focused on the G_ci_(α) relationship for Mode I, Mode II, and Mixed-Mode I/II loadings only. This relationship was investigated experimentally, taking to a large extent advantage of the concepts presented in ASTM D5528, D7905, and D6671 standards [26,27,28]; i.e., beam specimens were used. However, the actual design of the specimens (excluding the overall specimen dimensions) and the details of the data reduction procedures differed from that recommended by the mentioned standards.

**Figure 4:**
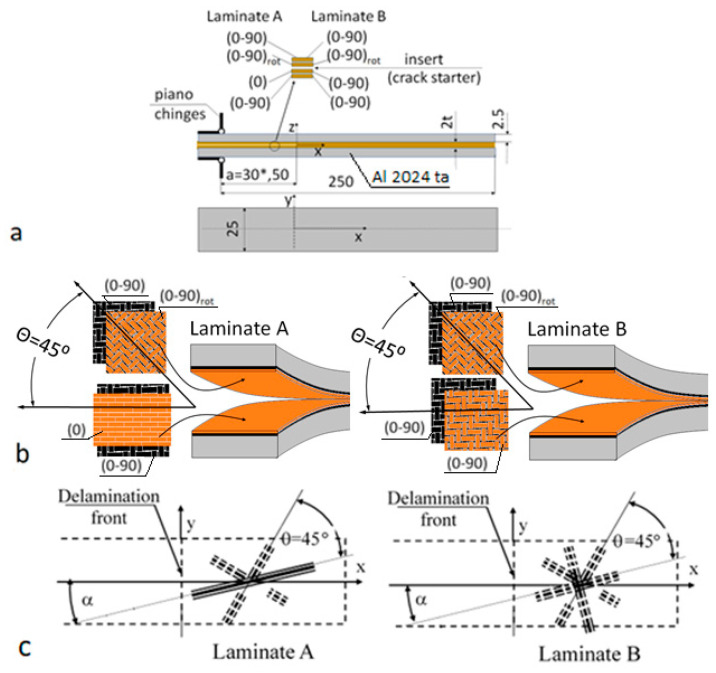
Hybrid beam specimen: (**a**) overall dimensions and design; (**b**) reinforcement arrangement; (**c**) definition of α.

Two laminates designated A and B were considered, as shown in Figure 4. Laminate A was composed of UD and symmetric fabric (2 × 2 twill), and θ = 45°. Laminate B was composed of symmetric fabrics only, but as in the previous case, θ = 45°. The laminates were tested for Ψ = 0° (Mode I), Ψ = 30°, 60° (Mixed Mode I/II), and for Ψ = 90° (Mode II) where Ψ was the phase angle (1).

(1) $$\Psi =\mathrm{atan}\sqrt{\frac{G_{\mathrm{II}}}{G_{I}}}$$

The test results showed that the variation in α exerted a statistically significant effect on the initiation values of the SERR, Gci, for both the laminates.

## 2. Materials and Methods

### 2.1. Materials

The considered laminates were reinforced with four layers of glass fiber fabric, (supplied by the Havel Composites PL). The fabrics were impregnated with Ep53/Z1 epoxy resin-hardener system, (the resin over hardener mass ratio was 10/1), supplied by the Sarzyna Chemicals. Glass fabrics were chosen for a practical reason. Such reinforcement is still very common and broadly used in the case of hand wet layup. Furthermore, manufacturing flaws such as disbonds (Figure 1) are often created if such a manufacturing process is applied and can result in delamination propagation.

The laminates were composed of four layers. Two inner layers were essential and needed to create the desired IPO. Two external layers were added for manufacturing reasons to facilitate the adhesive bonding of the inner layers to the metal facings. The applied reinforcement layups were as follows (Figure 4):

Laminate A

(0–90)/(0–90)_rot_//(0)/(0–90)

Laminate B

(0–90)/(0–90)_rot_//(0–90)/0–90),

where (0–90) stands for symmetric fabric, (0–90)_rot_ stands for the same fabric but rotated by 45° relative to the previous one; (0) stands for unidirectional fabric, and “//” indicates the delamination locations. Table 1 shows the relevant mechanical properties of the materials used for manufacturing the specimens.

### 2.2. Method

The modified, hybrid beam specimens were used to determine G_ci_ values for Mode I, Mode II, and Mixed-Mode I/II loadings. The overall dimensions and the design of the specimens are shown in Figure 4. For each combination of Ψ and α, eight specimens were tested.

#### 2.2.1. Issue of the Specimen Design

The major problem appearing in the intended tests and the beam specimens that had to be used was the inevitable lack of symmetry in xz and xy planes of the specimens and resulting deformation and stress–strain couplings, causing undesired loading modes. For example, in the case of DCB (Mode I) tests, the lack of symmetry in the xy plane results in an unwanted Mode II loading component, and the lack of symmetry in the xz plane results in coupling between normal stress and shear strain, inducing a Mode III component. One should notice that for the ENF test, a pure Mode II condition never occurs over the whole front length, even if specimens are perfectly symmetric, since the G_III_ component is always present next to the sides of a beam. Unfortunately, the complete removal of the mentioned couplings is not possible. Nevertheless, they can be reduced by modifying the design of the specimen, e.g., by making the investigated laminates thin and backing them with much thicker and stiffer isotropic metal strips, as shown in Figure 4 [23] or by selecting a particular layup design resulting in a quasi-isotropic laminate [9,29]. In the case of the presented research, the former was chosen because the latter would result in too high specimen stiffness due to the large number of reinforcement layers needed to produce sufficiently quasi-isotropic laminate; furthermore, the fabrication of such specimens would be more laborious.

##### Analysis of Coupling Coefficients

The general procedure leading to the reduction of the mentioned couplings is a reduction of the Dc (2) and Bt (3) coefficients [30,31].

(2) $$D_{c}=\frac{D_{12}^{2}}{D_{11}D_{22}}$$

(3) $$B_{t}=\frac{D_{16}}{D_{11}}$$

In (2) and (3), the D_11_, D_22_, D_12_, and D_16_ are components of the bending stiffness matrix, and their indexing follows generally accepted convention used, e.g., in [32].

**Figure 5:**
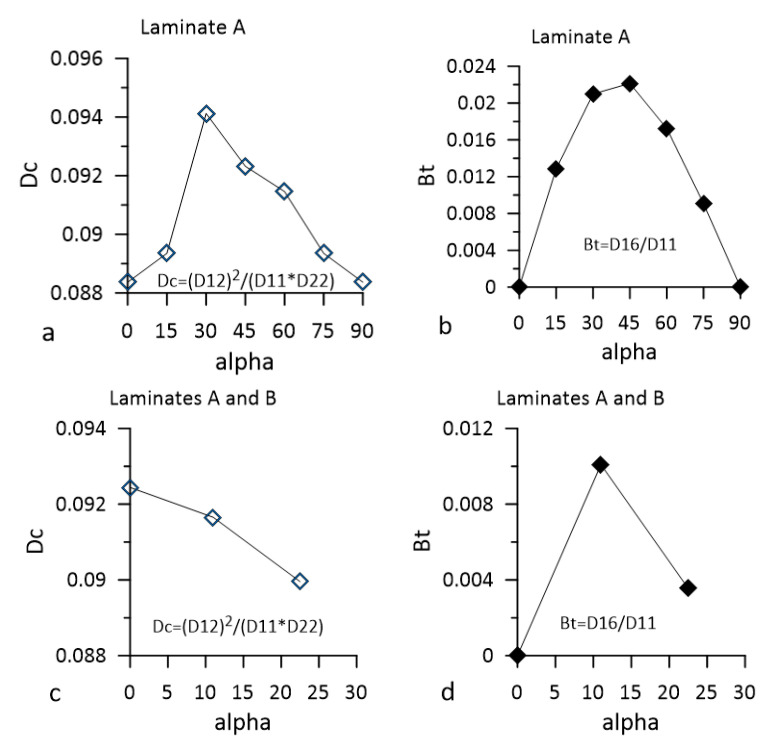
Dc and Bt coefficient calculated for the used hybrid beam specimens. (**a**,**b**) The lower branches of the specimen representing Laminate A; (**c**,**d**) upper branches of the specimens representing Laminates A and B.

It was suggested in [33] that D_c_ should be not higher than 0.25 and the acceptable of B_t_ ≤ 0.0315. The D_c_ and B_t_ values obtained for the specimens used in the presented research are shown in Figure 5 and were lower than those in [33].

In addition, the hybrid beam specimens of all the considered reinforcement arrangements were modeled using the ANSYS [34] commercial FE code to assess more precisely the “residual” coupling effects in terms of “undesirable” loading mode components. The ratios of the SERR components over the total value of the SERR were calculated for each reinforcement arrangement prior to the testing. The Modified Crack Closure Integral method [35] was used to determine the SERR components.

##### Finite Element Analysis of the Hybrid Specimens

**Figure 6:**
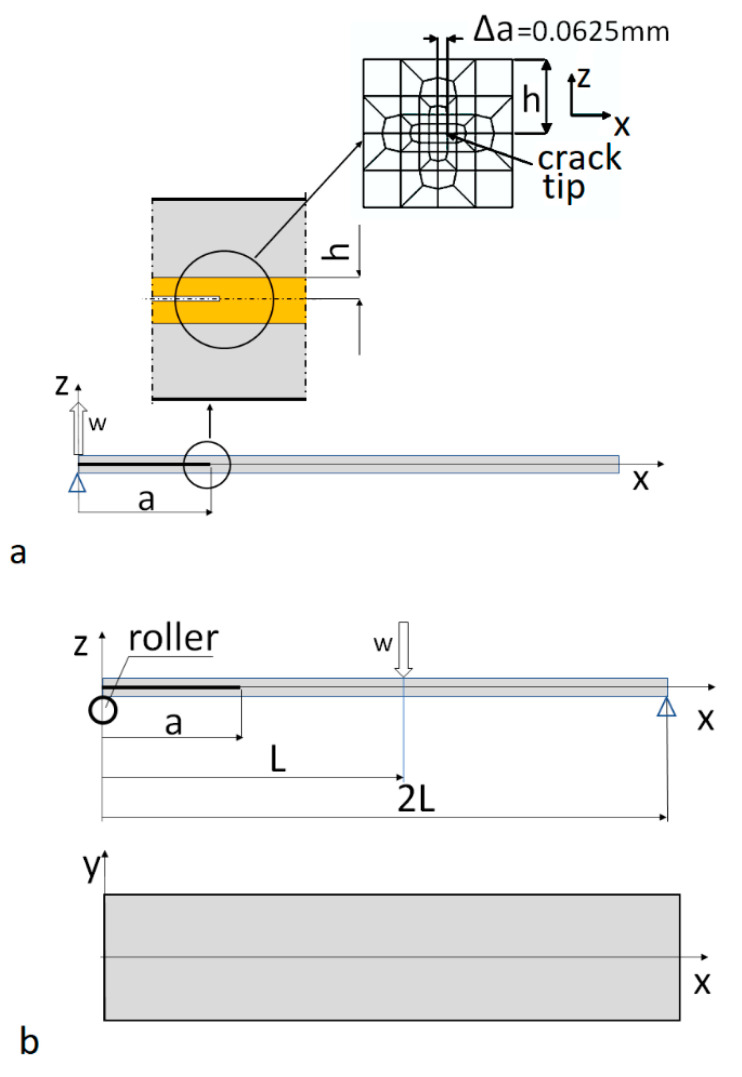
Modeled structures: (**a**) Mode I test; (**b**) Mode II test. Mesh design in the vicinity of the delamination front is shown in the xz plane. It was the same for both models. The h dimension represents the thickness of two reinforcement layers.

To analyze the SERR components’ distributions, a 3D FE model was built using the ANSYS commercial code [34]. The mesh design in the vicinity of the delamination front over the laminate thickness, 2 h = 1 mm, is shown in Figure 6. The metal facings and laminate were modeled with SOLID185 elements (Homogeneous Structural Solid, KEYOPT(3) = 0). The SERR components were calculated using the Modified Crack Closure Integral (MCCI) method [35]. To calculate P_x_, P_y_, and P_z_ components of the forces needed to close the crack of length, Δa = 0.0625 mm, three COMBIN14 elements with the appropriate freedom degrees (KEYOPT(2) = 1,2,3) were used for each node located at a distance, Δa, from the crack tip. To prevent penetration of the delamination faces, the surface-to-surface contact pair elements CONTA173 and TARGET170 were applied where necessary. In addition, the same contact elements were applied at the roller–specimen interface (Mode II model) to allow for out-of-plane deformation of the specimen branches.

Boundary conditions:

Mode I model (DCB test)

for the edge passing through points x = 0, y = ±b/2, z = h,

u = v = 0, w = 4 mm,

for the edge passing through points x = 0, y = ±b/2, z = -h

u = w = v = 0,

Mode II model (ENF test)

for x = L, y = −b/2, z = h,

v = 0

for the edge passing through points x = 2 L, y = ±b/2, z = -h

w = 0

for the edge passing through points x = L, y = ±b/2, z = h

u = 0, w = −3 mm

and for the roller all degrees of freedom were set to zero

#### 2.2.2. Specimen Manufacturing

First, 2.5 mm thick, 260 × 150 mm 2024 ta Al alloy plates were chemically cleaned using the pickling process. Next, one side of each plate was covered with BWF primer and cured. The fabrics were stacked and impregnated at the primer-treated surface of one of the metal plates using the wet layup manufacturing technique and then covered with the other metal plate. To facilitate delamination propagation, crack starters made of 15 μm thick Teflon foil were inserted between the reinforcement layers that were to be delaminated.

Laminate A was reinforced with INTERGLAS 92125 (2 × 2 twill) and INTERGLAS 92145 (UD) glass fabrics of areal weights 280 g/m^2^ and 220 g/m^2^, respectively, while Laminate B was reinforced only with INTERGLAS 92125 fabric. The fabrics were impregnated with the EPIDIAN 53 and Z1 hardener epoxy resin system, which was cured at room temperature to avoid thermal stress. The complete setups were vacuum cured at 20 °C for 6 h. The nominal fiber mass fraction was 0.5. Next, the plates were cut into 250 × 25 mm specimens using a slitting saw. The hinges were glued to the metal facings with glue suitable for curing at room temperature as well.

#### 2.2.3. Testing

**Figure 7:**
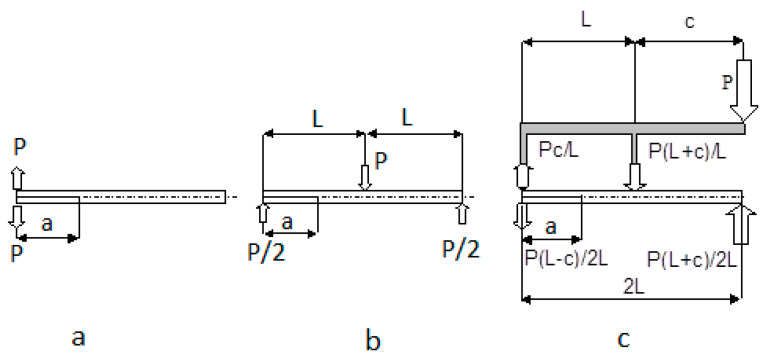
Loading schemes applied in the case of: (**a**) Mode I (ASTM D5528); (**b**) Mode II (ASTM D7905); (**c**) Mixed-Mode I/II (ASTM D6671) tests.

The loading schemes followed the ASTM D5528, D7905, and D6671 standards’ recommendations and are depicted in Figure 7. The laminates were tested for Ψ = 0° (Mode I), Ψ = 45°, 71° (Mixed Mode I/II), and for Ψ = 90° (Mode II).

The tests were carried out at room temperature under displacement-controlled conditions. The crosshead speed was 5 mm/min for Mode I tests and 1 mm/min for Mode II and Mixed-Mode I/II tests. The maximum load read from the load–displacement plots was used for calculating G_ci_ (subscript “i” stands for initiation). The simple beam theory was applied for all calculations. For Mode I and Mode II loadings, G_ci_ values were computed using Equations (4) and (5), and for Mixed-Mode I/II loadings, G_ci_ values were computed using Equations (6) and (7). The average values of the data provided in Table 2 were used to determine the correction factor χ. The values in this table were calculated for α = 0 according to the Classical Laminate Theory (CLT) and assumed to be effective moduli. One should mention that these values were approximated ones in the sense of the CLT because the sublaminates making the trunks and branches of the specimens were not symmetric, and A_16_ and A_26_ were not equal to 0; nevertheless, they were small.

(4) $$G_{\mathrm{Ic}}=\frac{3\mathrm{Pf}}{2\mathrm{Ba}}$$

(5) $$G_{\mathrm{IIc}}=\frac{9a^{2}\mathrm{Pf}}{2B\left(2L^{3}+3a^{2}\right)}$$

(6) $$G_{I}^{m}=\frac{12P^{2}{\left(3c-L\right)}^{2}}{16B^{2}h^{3}L^{2}E_{L}}{\left(a+\chi h\right)}^{2}$$

(7) $$G_{\mathrm{II}}^{m}=\frac{9P^{2}{\left(3c+L\right)}^{2}}{16B^{2}h^{3}L^{2}E_{L}}{\left(a+0.42\chi h\right)}^{2}$$

where (see Figure 6)

a—initial crack length;

B—specimen width;

c—lever arm;

E_L_—longitudinal modulus;

f—displacement of a loading point;

L—half of a specimen length;

P—load;

and

$$\chi =\sqrt{\frac{E_{L}}{G_{\mathrm{Lh}}}\left[3-2{\left(\frac{\Gamma }{1+\Gamma }\right)}^{2}\right]}$$

$$\Gamma =1.18\frac{\sqrt{E_{L}E_{T}}}{G_{\mathrm{Lh}}}$$

where

E_T_—transverse modulus;

G_Lh_—shear modulus in the plane parallel to the specimen thickness and length directions.

## 3. Results

### 3.1. Results of the FE Analysis

**Figure 8:**
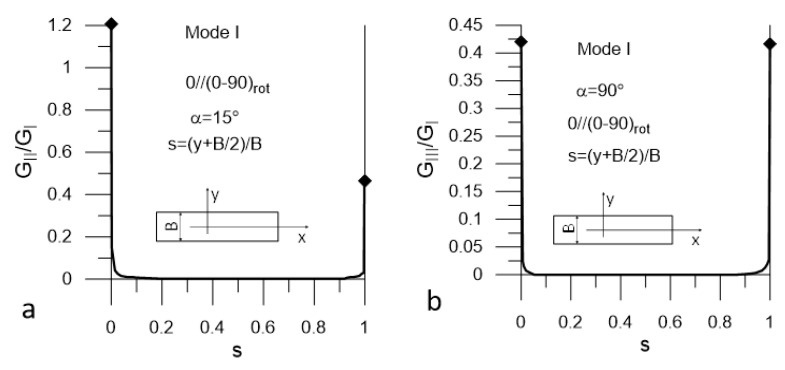
Mode I loading; (**a**) G_II_/G_I_ (s) ratio and (**b**) G_III_/G_I_ ratio for Laminate A.

**Figure 9:**
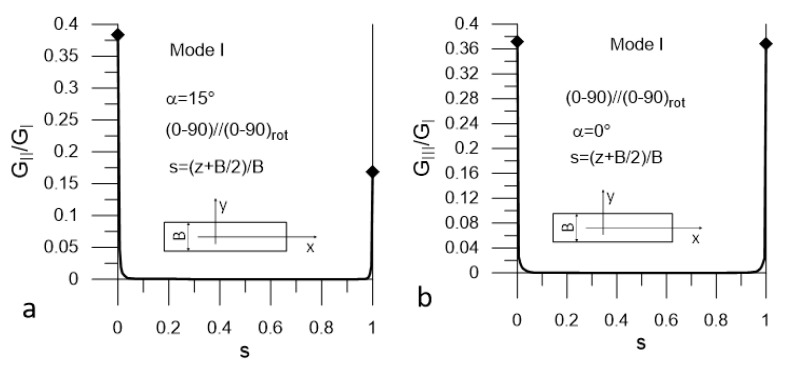
Mode I loading; (**a**) G_II_/G_I_ (s) ratio and (**b**) G_III_/G_I_ (s) ratio for Laminate B.

**Figure 10:**
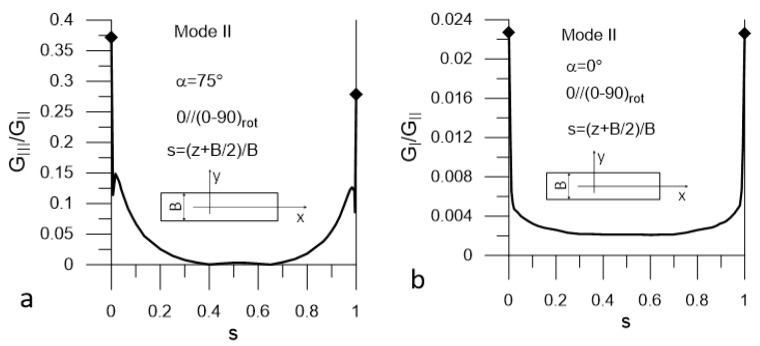
Mode II loading; (**a**) G_III_/G_II_ (s) ratio and (**b**) G_I_/G_II_ (s) ratio for Laminate A.

**Figure 11:**
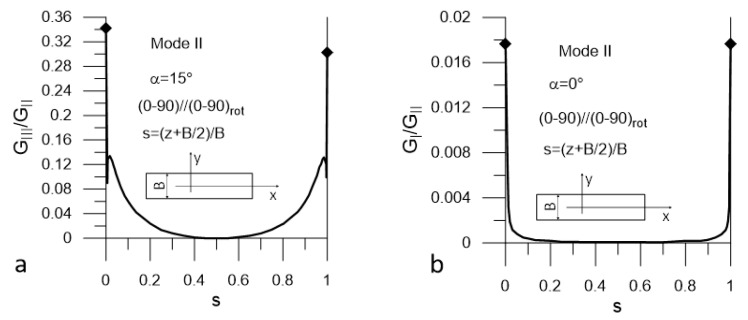
Mode II loading; (**a**) G_III_/G_II_ (s) ratio and (**b**), G_I_/G_II_ (s) for Laminate B.

The presented plots are limited to these corresponding to the most unfavorable values of α = α_uf_. The plots in Figure 8 and Figure 9 present distributions of G_II_/G_I_ and G_III_/G_I_ ratios for Mode I loading (DCB test) for Laminate A and Lamianta B, respectively. The plots in Figure 10 and Figure 11 present distributions of G_I_/G_II_ and G_III_/G_II_ ratios for Mode II loading (ENF test) for Laminate A and Laminate B, respectively. Under Mode I loading, for both laminates, the G_II_ and G_III_ components were present. In the case of Laminate A, the maximum contribution of G_II_ corresponded to α_uf_ = 15° and the maximum contribution of G_III_ corresponded to α_uf_ = 90°; see Figure 8. For Laminate B, the maximum contribution of G_II_ corresponded to α_uf_ = 15° and the maximum contribution of G_III_ corresponded to α_uf_ = 0°, as shown in Figure 9. Under Mode II loading, components G_I_ and G_III_ were present. For Laminate A, the maximum contribution of G_I_ occurred for α_uf_ = 0° and that of G_III_ occurred for α_uf_ = 75°, as shown in Figure 10. For Laminate B, the maximum contribution of G_I_ occurred for α_uf_ = 0° and of G_III_ for α_uf_ = 15°, as shown in Figure 11.

It could be noticed that under Mode I loading, for both laminates, the maximum values of the G_II_/G_I_ and G_III_/G_I_ ratios were relatively high next to the specimen edges and equaled to 1.2 and 0.42 respectively (Figure 8 and Figure 9). However, they dropped to zero at a very short distance from the edges. In the case Mode II loading for both laminates, the G_I_/G_II_ and G_III_/G_II_ ratios (Figure 10 and Figure 11) were lower than those of G_II_/G_I_ and G_III_/G_I_; however, for both laminates, the G_III_ component contributed to the total value of the SERR over a substantial part of the delamination front (Figure 10a and Figure 11a). For Laminate A, the G_I_ component was present over the entire length of the crack front, but the ratio of G_I_/G_II_ was very low (Figure 10b). At the specimen edges, it reached 0.025 and sharply dropped to about 0.0025 for the remaining part of the delamination front. For Laminate B, the maximum value of G_I_/G_II_ did not exceed 0.02 in the vicinity of the edges and decayed to zero over a short distance (Figure 11b).

Similar analysis concerning Mixed-Mode I/II loading is presented in [14]. It was found out that for the analyzed mixite ratios, the “undesired” loading modes were also limited to small sections of the delamination fronts next to the specimen edges.

### 3.2. Experimental Results

**Figure 12:**
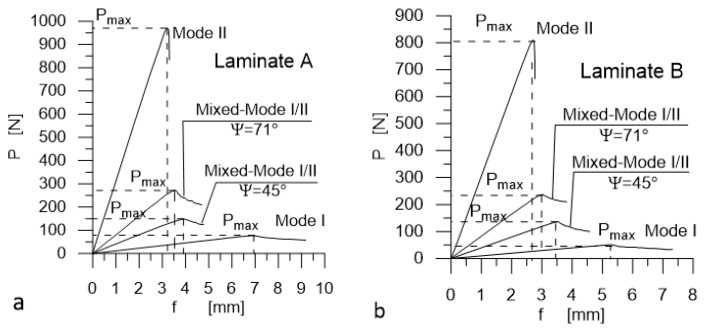
Examples of typical load–displacement plots; (**a**) for Laminate A and (**b**) for Laminate B.

**Figure 13:**
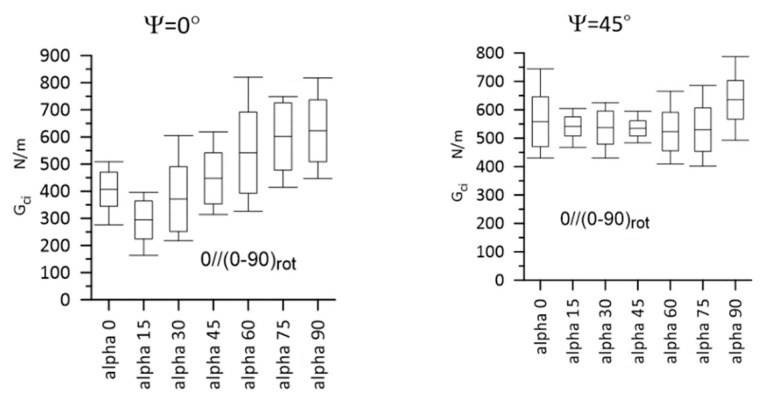
Gci values for Laminate A, for Ψ = 0° and Ψ = 45°. Statistically, a significant difference was detected for Ψ = 0°, for α = 0° vs. α = 90°; α = 15° vs. α = 60°; α = 30° vs. α = 75°; α = 30° vs. α = 90°. The whiskers limit the extreme values, the middle bars represent the mean values, and the boxes represent the standard deviations.

**Figure 14:**
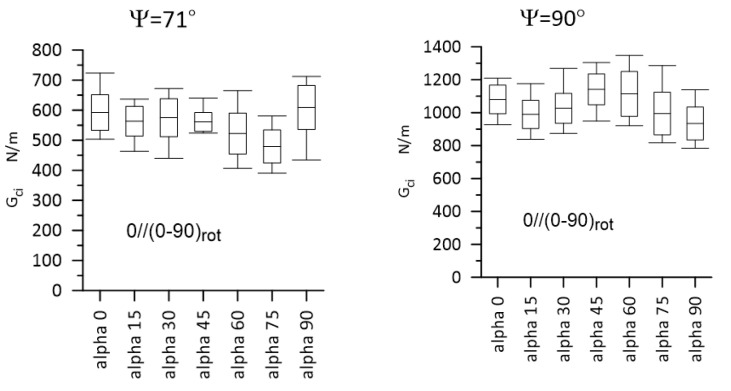
Gci values for Laminate A, for Ψ = 71° and Ψ = 90°. Statistically significant differences were detected for Ψ = 71°, for α = 0° vs. α = 75°; α = 75° vs. α = 90° and for Ψ = 90°, for α = 45° vs. α = 90°. The whiskers limit the extreme values, the middle bars represent the mean values, and the boxes represent the standard deviations.

**Figure 15:**
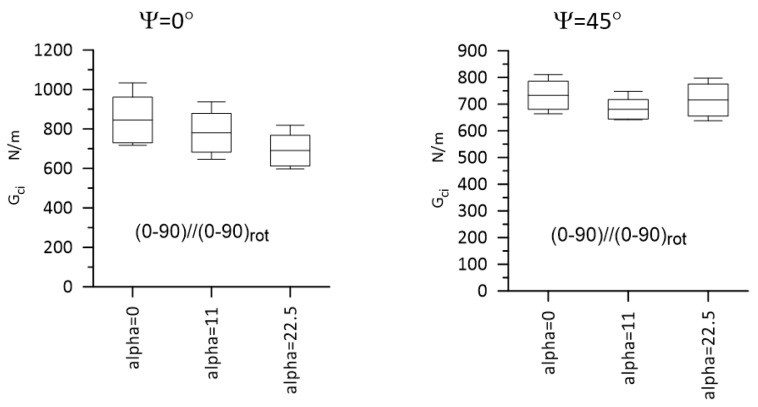
Gci values for Laminate B for Ψ = 0° and 45°. Statistically, a significant difference was detected for Ψ = 0°, for α = 0° vs. α = 22.5°. The whiskers limit the extreme values, the middle bars represent the mean values, and the boxes represent the standard deviations.

**Figure 16:**
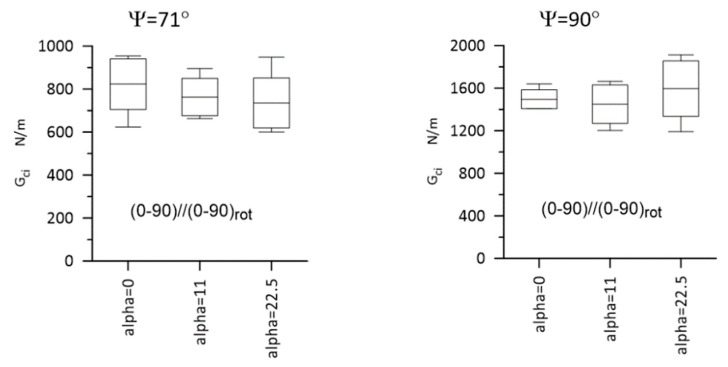
Gci values SERR for Laminate B, for Ψ = 71°, and for 90°. The whiskers limit the extreme values, the middle bars represent the mean values, and the boxes represent the standard deviations. Statistically significant differences were not detected.

Typical load–displacement plots corresponding to Mode I, Mode II, and Mixed-Mode I/II (Ψ = 45° and 71°) loadings are shown in Figure 12. The most important experimental results are presented in Figure 13 and Figure 14 for Laminate A, and for Laminate B, they are presented in Figure 15 and Figure 16. The whiskers limit extremes of the results of each test, the middle bars represent the mean values, and the boxes represent the standard deviations.

## 4. Discussion

The results displayed large scatter, which made the interpretation of them difficult. For this reason, statistical analysis was carried out using a one-way ANOVA test to check whether the differences between the means (DBM) were of statistical significance. In addition, to discriminate among the means, the Fisher’s least significant difference (LSD) test was used [36]. Prior to ANOVA tests, the data were checked for normality applying the Shapiro–Wilk test [37]. It showed that the differences between the data samples and the normal distributions were not big enough to be statistically significant. Therefore, it was assumed that the data were normally distributed. Next, the data were tested for homogeneity of variances using Levene’s test [38]. The results showed that the null hypothesis of homogeneity of variance could be accepted. The results of Shapiro–Wilk and Levene’s tests allowed for running one-way ANOVA tests. In the case of the Fisher test, there was a 5.0% risk of calling each pair of means significantly different when the actual difference equaled 0. The dependence of Gci on α was found out for both laminates. However, it was not equally strong, and it turned out that it was the loading mode and the interfacial ply arrangement sensitive. Statistically significant differences detected in the case of Laminate A for Mode I, Mixed-Mode I/II, and Mode II loadings are given in Table 3.

Statistically significant differences detected in the case of Laminate B are given in Table 4. They occurred for Mode I and Mixed-Mode I/II loading for Ψ = 45° only.

One could notice that for Laminate A and Mode I loading, Gic increased with α, while for Mode II loading, it decreased. For Laminate B and Mode I loading, Gic decreased with α, while for Mode II, it increased; however, it was found out that this increase was statistically insignificant. The stronger dependence of Gci on α for Laminate A than for Laminate B could be attributed to the less “isotropic” interfacial reinforcement arrangement of the former.

Below, for comparison purposes, the results of the most relevant experimental works that were found [10,23,24] regarding the presented research are shortly described. Unfortunately, this comparison, as regards the research presented by the author, may have an important shortcoming since papers [10,23,24] concern mainly laminates reinforced with UD tapes and impregnated with different resins.

**Figure 17:**
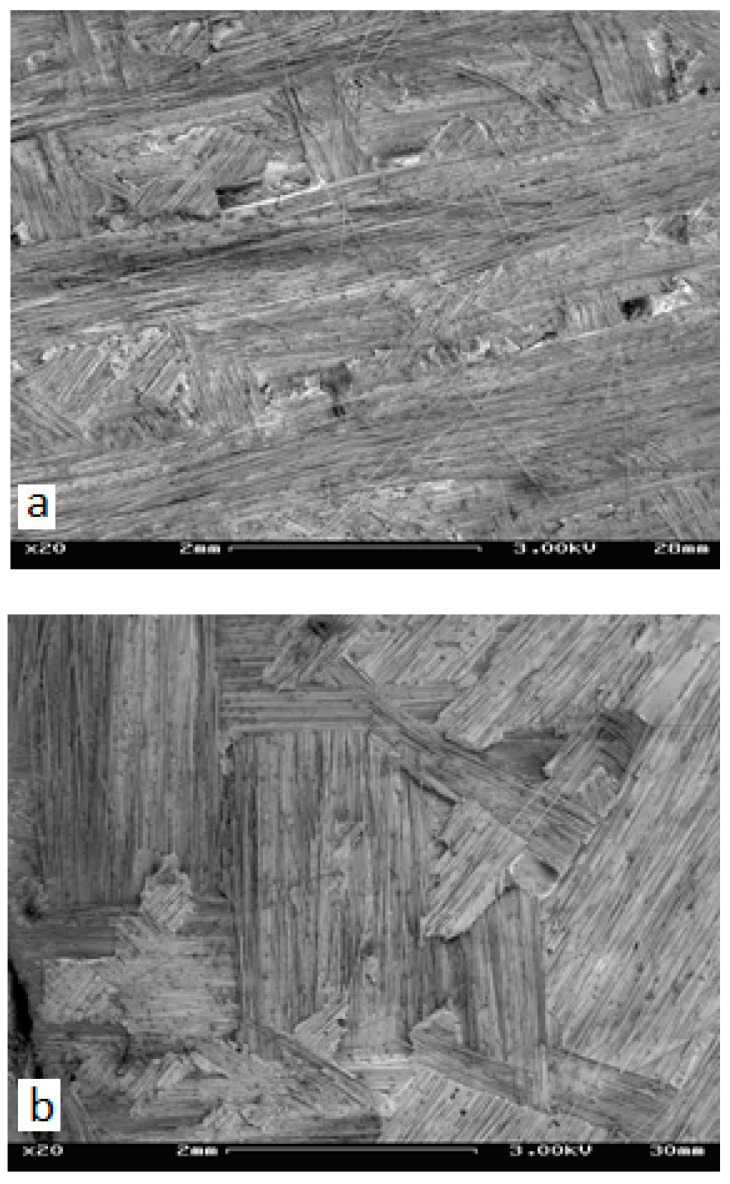
The complex morphology of the delamination surface: (**a**) Laminate A and (**b**) Laminate B.

Although the investigations presented in [10] did not focus on the Gci vs. α relationship, in principle, one could find out certain information concerning the Gci vs. α relationship. One could extract Gci for Mode I, for θ = 60° (θ_1_ = 30°, θ_2_ = −30°) and α = 30°, and for θ = 90° (θ_1_ = 45°, θ_2_ = −45°) and α = 45°. In both cases, an increase in Gci with α was reported. The dependence of Gci on α was also reported in [23] and [20] for α = 0°, 15°, 30°, and 45°. In [23], results were presented for Mode I and Mode II loadings, and θ = 90° (θ_1_ = 45° and θ_2_ = −45°). Under Mode I loading, a slight increase in Gci with α was reported, while under Mode II loading, the relationship was opposed. In [24], results were presented for Mode I and Mode II loadings, for θ = 45° (θ_1_ = 22.5°, θ_2_ = −22.5°) and θ = 90° (θ_1_ = 45°, θ_2_ = −45°). For θ = 45° and Mode I and Mode II loadings, the maximum values of Gci were reported for α = 0° and α = 45°. These values were the same for each loading mode. For θ = 90°, under Mode I loading, the Gci values initially decreased to reach the minimum for α = 15° and then increased monotonically to reach the maximum for α = 45°. Under Mode II loading, Gci increased with α to reach the maximum for α = 30° and then decreased. Based on the above, one can learn that the experimental results concerning the Gci(α) relationship were not equivocal, and they differed from laminate to laminate. Regarding the results obtained by the author, one could learn that (i) loading modes affected the Gci(α) relationship, and (ii) for all loading modes, the Gci(α) relationship was more pronounced for the less “isotropic” interface (Laminate A) than for the more “isotropic” one (Laminate B), as shown in Figure 17.

In-depth investigation of the reasons for such a variation of G_ic_ with α would require very extensive fractographic studies, being a problem all by itself. In the frame of the presented research, it was possible to perform only very limited fractographic investigations. The results showed high complexity of the delamination surface morphology, as shown in Figure 17.

**Figure 18:**
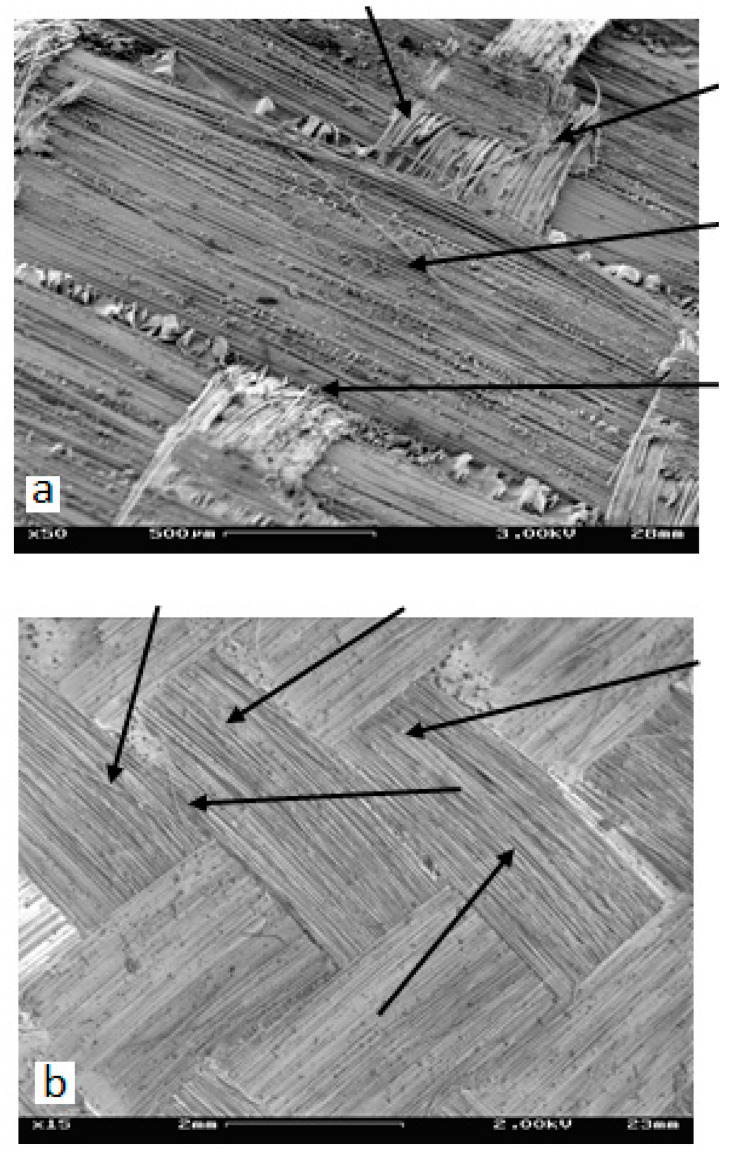
A small number of broken fibers (pointed out by arrows) were located at the strand periphery, indicating that the delamination roughly followed the strands envelope and did not penetrate the strands: (**a**) Laminate A, (**b**) Laminate B.

At higher magnification, one could notice that the penetration of the strands by the propagating delamination was limited to the most external fibers of strands and roughly followed the strands’ envelope, as shown in Figure 18, which was not planer. As a result of this, the Mixed-Mode conditions of various mode mixity, differing from the global ones, arose locally at the strand’s slopes. Due to characteristic fracture features [39,40] associated with a particular loading mode, one could detect the local presence of Mode I and Mode II loadings, as well as the presence of mixity of both loading modes. These local loading modes were associated with the local presence of manifold fracture mechanisms contributing to various degrees to the development of delamination.

**Figure 19:**
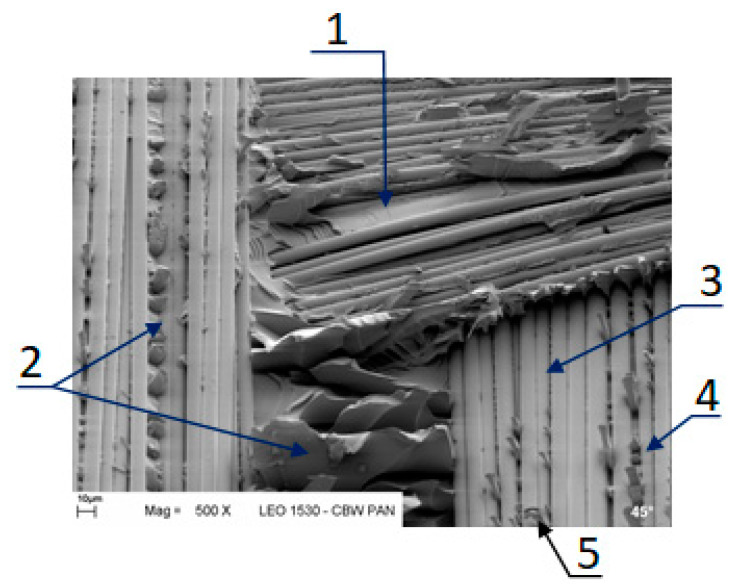
Local fracture modes and associated fracture mechanisms: (1) flat resin fracture surface between fibers indicate local Mode I loading, (2) resin cups indicate Mode II loading, (3) region of the backward bent strand-bear fibers indicate Mode I loading, (4) region of the strand parallel to the left-hand strand-caps indicated local Mode II loading, (5) fractured fiber.

Figure 19 provides some examples of these mechanisms. These include (i) a cohesive fracture of the matrix between the strands and fibers resulting in a flat resin surface, which was marked 1, or in resin cups, marked 2 and 4, (ii) the adhesive fracture at the fiber–matrix interface resulting in a bare fiber surface, which was marked 3, and (iii) the fracture of fibers, which was marked 5. (Detailed discussion of this issue can be found in [41] The extent of each mechanism and its contribution to the delamination growth varied with location and presumably with α, which would be responsible for the variation of G_ic_ with α. Unfortunately, very extensive fractographic investigations would be necessary to verify this hypothesis, as was mentioned. Such studies were out of the scope of this research; nevertheless, they shall be a topic of future investigations.

## 5. Conclusions

The dependence of Gci on α was investigated under Mode I, Mode II, and Mixed-Mode I/II loadings for two different interfacial fiber arrangements. Delaminations were located at the interface of UD and symmetric fabrics (Laminate A) and at the interface of symmetric fabrics (Laminate B). Occurrence of the mentioned dependence was found out for both interfacial fiber arrangements; however, it was not equally strong. It was affected by the loading modes and the interfacial fiber arrangement.

## Figures and Tables

**Table 1:** Mechanical properties of the materials (laminates and backing metal sheet) used for manufacturing the specimens.

Material	E_11_ N/mm^2^	E_22_ N/mm^2^	G_12_ N/mm^2^	ν_12_
Laminate reinforced with Interglas 92125	23,000 *	23,000 *	4200 **	0.13 *
Laminate reinforced with Interglas 92145	32,000 *	8400 *	3500 **	0.33 *
Al 2024 ta	E_11_ = E_22_ = E = 72,000		27,700	0.3

* in-home tests carried out following ASTM D3039 standard. ** in-home tests carried out following ASTM D3518 standard.

**Table 2:** Effective mechanical properties of specimens.

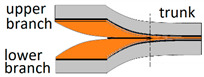		E_x_ N/mm^2^	E_y_ N/mm^2^	G_xy_ N/mm^2^	ν_xy_
Beam specimen encapsulating Laminate A	Trunk	63,870	62,950	2400	0.3
	Upper branch	59,930	59,930	2314	0.3
	Lower branch	62,680	59,230	2200	0.29
Beam specimen encapsulating Laminate B	Trunk	63,520	63,520	2403	0.3
	Upper branch	59,930	59,930	2314	0.3
	Lower branch	61,440	61,440	2214	0.28
average		61,895	61,167	2308	0.295

**Table 3:** Laminate A: statistically significant differences.

Ψ	α_i_ vs. α_j_	DBM	LSD
0°	α = 0° vs. α = 60°	135	131
	α = 0° vs. α = 75°	195	131
	α = 0° vs. α = 90°	216	131
	α = 15° vs. α = 45 °	153	131
	α = 15° vs. α = 60 °	248	131
	α = 15° vs. α = 75 °	307	131
	α = 15° vs. α = 90°	329	131
	α = 30° vs. α = 60°	171	131
	α = 30° vs. α = 75°	231	131
	α = 30° vs. α = 90°	252	131
	α = 45° vs. α = 75°	155	131
	α = 45° vs. α = 90°	176	131
45° (G_II_/G_I_ = 1)	α = 15° vs. α = 90°	94	78
	α = 30° vs. α = 90°	98	78
	α = 45° vs. α = 90°	101	78
	α = 60° vs. α = 90°	112	78
	α = 75° vs. α = 90°	105	78
71° (G_II_/G_I_ = 1.7)	α = 0° vs. α = 75°	113	72
	α = 15° vs. α = 75°	84	72
	α = 30° vs. α = 75°	96	72
	α = 45° vs. α = 75°	82	72
	α = 60° vs. α = 90°	87	72
	α = 75° vs. α = 90°	130	72
90°	α = 0° vs. α = 90°	146	130
	α = 15° vs. α = 45°	152	130
	α = 45° vs. α = 75°	147	130
	α = 45° vs. α = 90°	208	130
	α = 60° vs. α = 90°	180	130

**Table 4:** Laminate B: statistically significant differences.

Ψ	α_i_ vs. α_j_	DBM	LSD
0°	α = 0° vs. α = 22.5°	155	102
45° (G_II_/G_I_ = 1)	α = 0° vs. α = 15°	52.4	52.3

## Data Availability

Data are available on request.

## References

[1] Allix O., Leveque D., Perret L. (1998). Identification and forecast of delamination in composite laminates by an interlaminar interface model. Compos. Sci. Technol..

[2] Chou I., Kimpara I., Kageyama K., Ohsawa I., Martin R.H. (1995). Mode I and Mode II Fracture Toughness Measured between Differently Oriented Plies in Graphite/Epoxy Composites, Composite Materials: Fatigue and Fracture.

[3] Ozdil F., Carlsson L.A., Davis P. (1998). Beam analysis of angle-ply laminate end-notched flexure specimens. Compos. Sci. Technol..

[4] Ozdil F., Carlsson L.A. (1999). Beam analysis of angle-ply laminate DCB specimens. Compos. Sci. Technol..

[5] Polaha J.J., Davidson B.D., Hudson R.C., Piera C.A. (1996). Effects of Mode Ratio, Ply Orientation and Precracking on the Delamination Toughness of a Laminated Composite. J. Reinf. Plast. Compos..

[6] Pereira A.B., de Morais A.B. (2004). Mode I interlaminar fracture of carbon/epoxy multidirectional laminates. Compos. Sci. Technol..

[7] Pereira A.B., de Morais A.B. (2004). Mode II interlaminar fracture of glass/epoxy multidirectional laminates. Compos. Part A.

[8] Pereira A.B., de Morais A.B. (2006). Mixed mode I + II interlaminar fracture of glass/epoxy multidirectional laminates—Part 2: Experiments. Compos. Sci. Technol..

[9] Bin Mohamed Rehan M.S., Rousseau J., Fontaine S., Gong X.J. (2017). Experimental study of the influence of ply orientation on DCB mode-I delamination behavior by using multidirectional fully isotropic carbon/epoxy laminates. Compos. Struct..

[10] Rzeczkowski J., Samborski S., De Moura M. (2020). Experimental Investigation of Delamination in Composite Continuous Fiber-Reinforced Plastic Laminates with Elastic Couplings. Materials.

[11] Banks-Sills L., Ishbir C.H., Fourman V., Rogel L., Eliasi R. (2013). Interface fracture toughness of a multi-directional woven, Composite. Int. J. Fract..

[12] Chocron T., Banks-Sills L. (2019). Nearly Mode I Fracture Toughness and Fatigue Delamination Propagation in a Multidirectional Laminate Fabricated by Wet Layup. Phys. Mezomech..

[13] Banks-Sills L., Simon I. (2021). Comparison of calculations of energy release rates for DCB multi-directional laminate specimens. Int. J. Fract..

[14] Pichler N., Herraez M., Botsis J. (2020). Mixed-mode fracture response of anti-symmetric laminates: Experiments and modeling. Compos. Part B.

[15] Triki E., Zouari B., Jarraya A., Dammak F. (2013). Ply Orientations Effect in the Fracture Toughness of Mixed Mode Delamination in E-Glass/Polyester Woven Fabrics. Des. Modeling Mech. Syst..

[16] Bican I.E., Mete Onur Kaman M.O., Erdem S. (2020). Effect of fiber orientation on interfacial fracture toughness for adhesively bonded composite plates. J. Mech. Sci. Technol..

[17] Rzeczkowski J., Samborski S., Paśnik J. (2018). Experimental Investigation of Mechanically Coupled Composite Specimens in the ENF Test Configuration. Proceedings of the IOP Conference Series: Materials Science and Engineering, 2018.

[18] Ramji A., Xu Y., Grasso M., Yasaee M., Webb P. (2021). Effect of interfacial fibre orientation and PPS veil density on delamination resistance of 5HS woven CFRP laminates under mode II loading. Compos. Sci. Technol..

[19] Raimondo A., Urcelay Oca I., Bisagni C. (2021). Influence of interface ply orientation on delamination growth in composite laminates. J. Compos. Mater..

[20] Blondeau C., Pappas G., Botsis J. (2019). Influence of ply-angle on fracture in antisymmetric interfaces of CFRP Laminates. Compos. Struct..

[21] Gong Y., Zhang B., Zhao L., Zhang J., Hua N., Zhang C. (2019). R-curve behaviour of the mixed-mode I/II delamination in carbon/epoxy laminates with unidirectional and multidirectional interfaces. Compos. Struct..

[22] Hudișteanu I., Țăranu N., Isopescu D.N., Ungureanu D., Axinte A., Ghiga D.A. (2020). The influence of fibre orientation and of the adjacent layers on the delamination of laminated composites. Proceedings of the IOP Conference Series: Materials Science and Engineering, 2020.

[23] Rubbrecht P.H., Verpoest I. The development of two new test methods to determine the mode I and mode II fracture toughness for varying fibre orientation at the interface. Proceedings of the 38th International SAMPE Symposium and Exhibition.

[24] Hwang J.H., Lee C.S., Hwang W. (2001). Effect of Crack Propagation Directions on the Interlaminar Fracture Toughness of Carbon/Epoxy Composite Materials. Appl. Compos. Mater..

[25] Czarnocki P. (2000). Effect of reinforcement arrangement on distribution of GI, GII and GIII along fronts of circular delaminations in orthotropic composite plates. Eur. Struct. Integr. Soc..

[26] ASTM. D5528-01 (2017). Standard Test Method for Mode I Interlaminar Fracture Toughness of Unidirectional Fiber-Reinforced Polymer Matrix Composites.

[27] ASTM. D6671-01 (2017). Standard Test Method for Mixed Mode I—Mode II Interlaminar Fracture Toughness of Unidirectional Fiber-Reinforced Polymer Matrix Composites.

[28] ASTM. D7905 (2017). Standard Test Method for Determination of the Mode II Interlaminar Fracture Toughness of Unidirectional Fiber-Reinforced Polymer Matrix Composites.

[29] Vannucci P., Verchery G. (2002). A new method for generating fully isotropic laminates. Compos. Struct..

[30] Davidson B.D., Schapery R.A. (1988). Effect of Finite Width on Deflection and Energy Release Rate of an Orthotropic Double Cantilever Beam Specimen. J. Compos. Mater..

[31] Davidson B.D. (1990). An Analytical of Delamination Front Curvature in Double Cantilever Beam Specimens. J. Compos. Mater..

[32] Jones R.J. (1975). Mechanics of Composite Materials.

[33] Davidson B.D., Krüger R., König M. (1996). Effect of Stacking Sequence on Energy Release Rate Distributions in Multidirectional DCB and ENF Specimens. Eng. Fract. Mech..

[34] Ansys© Release 15.0. https://dokumen.tips/documents/ansys-mechanical-apdl-theory-reference-15pdf.html.

[35] Rybicki E.F., Kanninen M.F. (1977). A finite element calculations of stress intensity factors by a Modified Crack Closure Integral. Eng. Fract. Mech..

[36] https://www.easycalculation.com/statistics/fishers-lsd-calculator.php.com/statistics/fishers-lsd-calculator.phphttps://www.easycalculation.com/statistics/fishers-lsd-calculator.php.

[37] https://www.statskingdom.com/320ShapiroWilk.html.

[38] https://www.statskingdom.com/230var_levenes.html.

[39] Pruslow D. (1981). Some fundamental aspects of composite fractography. Composites.

[40] Pruslow D. (1986). Matrix fractography of fibre-reinforced epoxy composites. Composites.

[41] Czarnocki P. (2017). Fractography of interlaminar fracture of gf/epoxy laminates, reinforced with fabrics. Compos. Theory Pract..

